# Choice of radiotherapy modality for the combined treatment of non-small cell lung cancer with brain metastases: whole-brain radiation therapy with simultaneous integrated boost or stereotactic radiosurgery

**DOI:** 10.3389/fonc.2023.1220047

**Published:** 2023-09-22

**Authors:** Xiaotao Dong, Kunlun Wang, Hui Yang, Yan Li, Yanqi Hou, Jiali Chang, Ling Yuan

**Affiliations:** Department of Radiation Oncology Affiliated Cancer Hospital of Zhengzhou University, Zhengzhou, Henan, China

**Keywords:** brain metastasis, simultaneous integrated boost, stereotactic radiosurgery, non-small cell lung cancer, combined therapy, radiotherapy

## Abstract

**Purpose:**

To compare Whole-brain radiation therapy with simultaneous integrated boost (WBRT+SIB) to stereotactic radiosurgery (SRS)for non-small cell lung cancer (NSCLC)with brain metastases (BMs)in terms of overall survival (OS), intracranial progression-free-survival(iPFS), toxicity and objective response rate (ORR)

**Methods:**

A retrospective review was performed in our hospital of 90 patients diagnosed with NSCLC- BM who received either SRS (n = 48) or WBRT+SIB (n = 42) from January 2016 to January 2022. 76 (84.44%) patients received systemic drug therapy after radiotherapy, including chemotherapy(n=53), targeted therapy(n=40), immunotherapy(n=23), and anti-vascular drug therapy(n=45). OS and iPFS were estimated by the Kaplan-Meier method and compared using the log-rank test. Univariate and Multivariate analysis of the prognostic factors was performed using the Cox proportional hazard regression model.

**Results:**

The WBRT+SIB cohort had a longer median iPFS (20.0 versus (VS) 12.0 months, P = 0.0069) and a similar median OS (32.0 vs 28.0 months, P = 0.195) than the SRS cohort. Intracranial objective response rates in WBRT +SIB and SRS cohorts were 76.19% and 70.09%, respectively (P = 0.566). Disease control rates were 88.09% and 83.33%, respectively (P = 0.521). Multivariate analysis showed that WBRT+SIB is the only factor affecting iPFS(hazard ratio (HR):0.597 {95%confidence interval(CI):0.370-0.966}, P=0.035). Sex, Liver metastasis and Lymph node metastasis are risk factors for NSCLC-BM.

**Conclusion:**

In the context of systemic drug therapy, WBRT+SIB may have better intracranial local control than SRS in NSCLC-BM patients.

## 1 Introduction

Among the most common cancers, lung cancer ranks first in cancer-associated death worldwide. More than 80% of lung cancer patients are non-small cell lung cancer (NSCLC). Brain metastases (BMs) from NSCLC represent an unmet need of increasing relevance as their incidence is rising considerably. Early use of magnetic resonance imaging/positron emission tomography-computed tomography (MRI/PET-CT) and improvements in therapies for systemic disease and ageing populations are contributing factors to this increasing incidence. The treatment of NSCLC-BM patients was always the hotspot of study. Neurosurgical resection is usually reserved for patients with good performance status, low-burden oligometastatic disease, and controlled extracranial/primary disease. Radiotherapy and drug therapy remain the primary treatment for many BM patients (1). The conventional view is that anti-tumor drugs are subject to the central nervous system (CNS) barrier (blood-brain barrier/blood-tumor barrier). However, Several studies have shown that novel drugs, such as three generations-targeted drugs and immune checkpoint inhibitors (ICIs), can achieve effective therapeutic concentrations in the CNS (2, 3). In addition, Radiation has synergistic effects with the drugs mentioned above (4–9). However, the local control (LC) rate is still unsatisfactory when treated with Whole- Brain Radiation Therapy/Stereotactic radiosurgery (WBRT/SRS) alone. WBRT with Simultaneous Integrated Boost (SIB) can enhance the intracranial control more than WBRT (10, 11), and SIB has the biological advantage of dose fractionation. One of the critical unanswered questions in the BM therapy field is the choice of radiotherapy mode under the principle of drug combined with radiation. It is unclear whether WBRT + SIB can improve efficacy and reduce toxicity compared with SRS. This study aimed to research the efficacy of these two conventional radiotherapy modalities WBRT+SIB and SRS, and investigate the prognostic factors, providing a reference for establishing the best strategy for treating NSCLC-BM.

## 2 Materials and methods

The clinical data of NSCLC-BM patients who underwent radiotherapy in the Affiliated Cancer Hospital of Zhengzhou University from January 2016 to January 2022 were retrospectively analyzed. This study was approved by the ethics committees of the Affiliated Cancer Hospital of Zhengzhou University & Henan Cancer Hospital, Zhengzhou, China. Due to the retrospective nature of the study and because no patient specimens were used, the requirement for informed consent was waived by the ethics committees. The inclusion criteria were as follows:(1) all included patients were confirmed by pathological diagnosis with primary lung cancer. (2) brain metastases were confirmed by CT scan or MRI. (3) radiotherapy, including WBRT+SIB or SRS and (4) clinical data integrity. The exclusion criteria were as follows: (1) received BM resection (2) small cell lung cancer (3) meningeal metastases. Finally, 90 patients were enrolled in this study. We collected baseline characteristics about the patients, including age, gender, BM numbers and the longest diameter, clinicopathological type,BMI, Distant metastatic status other than the brain(Liver,Bone,Lymph node and Contralateral lung), Common geriatric diseases such as hypertension and Glycuresis, Karnofsky performance status (KPS), radiotherapy modality, extracranial metastasis status, and post-radiotherapy treatment including chemotherapy, targeted therapy, immunotherapy, and anti-angiogenic drugs therapy. Besides, radiotherapy dose, start and end time of radiotherapy, date of intracranial progression and date of death, and radiotherapy-related toxicity were also collected.

### 2.1 Radiotherapy strategy

Radiotherapy was administered using WBRT+SIB or SRS. Patients were placed in the supine position. The head was immobilized with a thermoplastic mask; Enhanced CT was performed to localize the scan from the cranial vault to the cricoid cartilage with a layer thickness of 2 mm. The localization images were transmitted to the ECLIPSE planning system and fused with brain MRI images. Outline the target area on the ECLIPSE system. The gross tumor volume (GTV) was the metastases visible on the image, the clinical target volume (CTV) was the whole brain, GTV and CTV were exenterated 2 mm as the planning gross tumor volume (P-GTV) and clinical gross tumor volume(P-CTV). Besides, Outline the relevant organs at risk (e.g., optic nerve, optic cross, eye, crystal, brainstem, hippocampus, etc.) Radiotherapy schedule: IMRT 6MV-Xray P-CTV: 30Gy/3Gy/10f, P-GTV: 45Gy/4.5Gy/10f. 5 treatments per week (Mon-Fri). The prescribed dose of SRS varies according to the longest BM diameter (16-24Gy).

### 2.2 Follow-up

Data was obtained from inpatient medical records, and follow-up data was obtained by contacting patients by phone, home visits, or questionnaires. A complete inpatient medical record was available for each patient. Clinical efficacy and adverse effects were evaluated, and the final results were based on the data from the last follow-up visit.

### 2.3 Endpoint

The primary endpoint was Overall Survival (OS) and Intracranial Progression-free Survival(iPFS), while the secondary endpoint was the objective intracranial response. Objective response rate (ORR) = the number of (CR+PR) cases/total cases × 100%, and disease control rate (DCR) = the number of (CR+PR+SD) cases/total cases × 100%. iPFS was the time from radiotherapy to intracranial progression or patient death. OS was defined as the time from the start of radiotherapy to death or the last follow-up (2023.01.01). Progression was defined as >20% increase in BM diameter or new BM on imaging brain CT/MRI according to RECIST 1.1 criteria. Imaging evaluation of brain CT/MRI was performed monthly until the third month and reviewed every three months afterwards.

### 2.4 Statistical analysis

R studio (version 4.2.3) and SPSS Statistics software, version 26.0 (IBM Corporation, Armonk, NY, United States), were used for the analysis in this study. The cardinality test was used to compare the two groups’ differences in categorical variables, objective response rates, and toxicity. Kaplan-Meier method was used to analyze the iPFS and OS of the two groups and plot survival curves, and the log-rank test was used for different assessments. Univariate and Multivariate analyses used COX proportional risk regression models to estimate prognosis-related independent factors. After univariate analysis, clinical factors with P < 0.20 were included in the Multivariate Cox proportional risk regression model for analysis, reporting hazard ratio (HR) and 95% confidence interval (CI). All tests were performed bilaterally. P < 0.05 was considered statistically significant.

## 3 Results

### 3.1 Baseline characteristics

**Figure 1:**
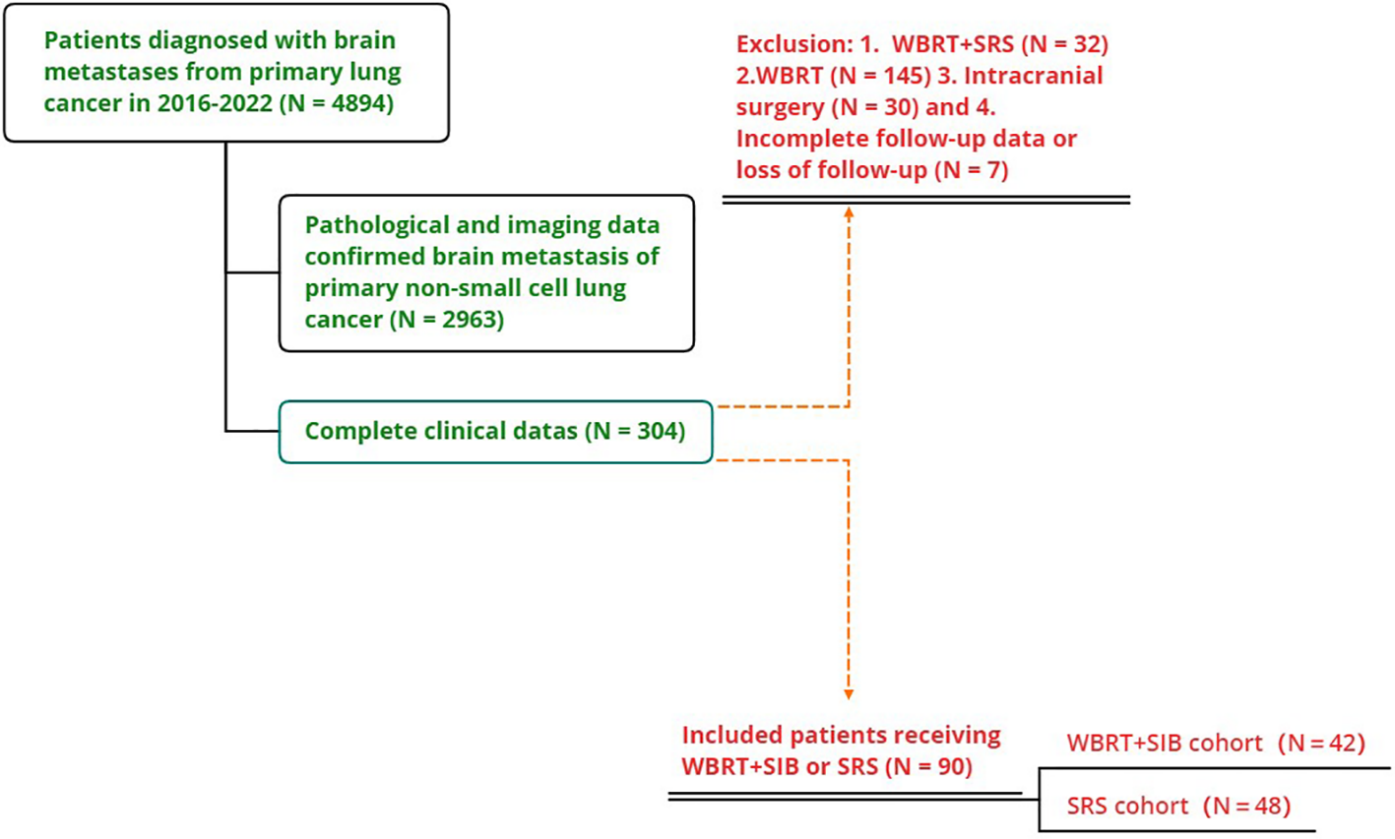
The flowchart of the database filtering process.

This study included 2963 patients with NSCLC-BM who were treated at the Affiliated Cancer Hospital of Zhengzhou University from January 2016 to January 2022, of whom 145 received WBRT, 32 received WBRT+SRS, 30 underwent neurosurgery, and 7 patients had missing data. Only 90 patients (42 underwent WBRT+SIB and 48 underwent SRS) were included in the study (**Figure 1**). The mean age of the total population was 60.1 years old (range 32-77 years),62 patients (68.89%) were ≤65 years old, 55 patients (61.11%) were male, 75 patients (83.33%) were adenocarcinoma and 40 cases had gene mutation (31 were EGFR+,7 were ALK+, and 2 were ROS1+), 73(81.11%) patients with KPS ≥70. The proportion of patients with single BM was significantly higher in the SRS group than in the WBRT+SIB group (16.67% vs 56.25%, P < 0.05). 76 patients (84.44%) received drug therapy after radiotherapy, of which 55 patients (61.11%%) received chemotherapy, 40 patients (44.44%) received targeted therapy, 23 patients (25.56%) received immunotherapy, and 45 patients (50.0%) received anti-angiogenic drugs. Other distant metastatic organs other than brain metastases, 35(38.9%) patients had liver metastases, 34(37.8%) had distant lymph node metastases, 27(30.0%) had contralateral lung metastases, and 27(30.0%) had bone metastases. All baseline patient characteristics are shown in **Table 1**.

**Table 1:** Patients characteristics.

	WBRT+SIB(N=42)	SRS(N=48)	Pvalue
Median age and range	59(32-77)	62(42-76)	
Age			
≤65	34(80.95%)	28(58.33%)	0.084
>65	8 (19.05%)	20(41.67%)	
Sex			0.773
Male	17(40.48%)	18(37.50%)	
Female	25(59.52%)	30(62.50%)	
BMI			0.756
≤23.9	36(85.71%)	40(83.3%)	
>23.9	6(14.29%)	8(16.7%)	
Number of BM			P<0.005
1	7 (16.67%)	27(56.25%)	
>1	35(83.33%)	21(43.75%)	
Diameter of the largest BM			0.245
≤3cm	34(80.95%)	43(89.58%)	
>3cm	8 (19.05%)	5 (10.42%)	
Histological status			0.257
Squamous cell	5(11.90%)	10(20.83%)	
Adenocarcinoma	37(88.10%)	38(79.17%)	
Surgery before RT			0.488
No	30(71.43%)	31(64.58%)	
Yes	12(28.57%)	17(35.42%)	
KPS			0.297
<70	6(14.29%)	11(22.92%)	
≥70	36(85.71%)	37(77.08%)	
Hypertension			0.215
No	19(45.24%)	28(58.33%)	
Yes	23(55.76%)	20(41.67%)	
Glycuresis			0.027
No	29(69.04%)	22(45.83%)	
Yes	13(30.96%)	26(54.17%)	
Liver metastasis			0.563
No	27(64.29%)	28(58.33%)	
Yes	15(35.71%)	20(41.67%)	
Lymph metastasis			0.706
No	27(64.29%)	29(60.42%)	
Yes	15(35.71%)	19(39.58%)	
Contralateral lung metastasis			0.782
No	30(71.42%)	33(68.75%)	
Yes	12(28.58%)	15(31.25%)	
Bone metastasis			0.461
No	31(73.81%)	32(66.67%)	
Yes	11(26.19%)	16(33.33%)	
Chemotherapy after RT			0.753
No	18(42.86%)	19(39.58%)	
Yes	24(57.14%)	29(60.42%)	
Target therapy after RT			0.571
No	22(52.38%)	28(58.33%)	
Yes	20(47.62%)	20(41.67%)	
Immunotherapy after RT			0.722
No	32(76.19%)	35(72.92%)	
Yes	10(23.81%)	13(27.08%)	
Anti-angiogenic drug therapy after RT			0.673
No	20(52.38%)	25(60.42%)	
Yes	22(47.62%)	23(39.58%)	

WBRT+SIB, Whole- Brain Radiation Therapy with Simultaneous Integrated Boost; SRS, Stereotactic Radiosurgery; BM, brain metastasis; RT, Radiotherapy; BMI, Body mass index.

### 3.2 Prognostic information

**Figure 2:**
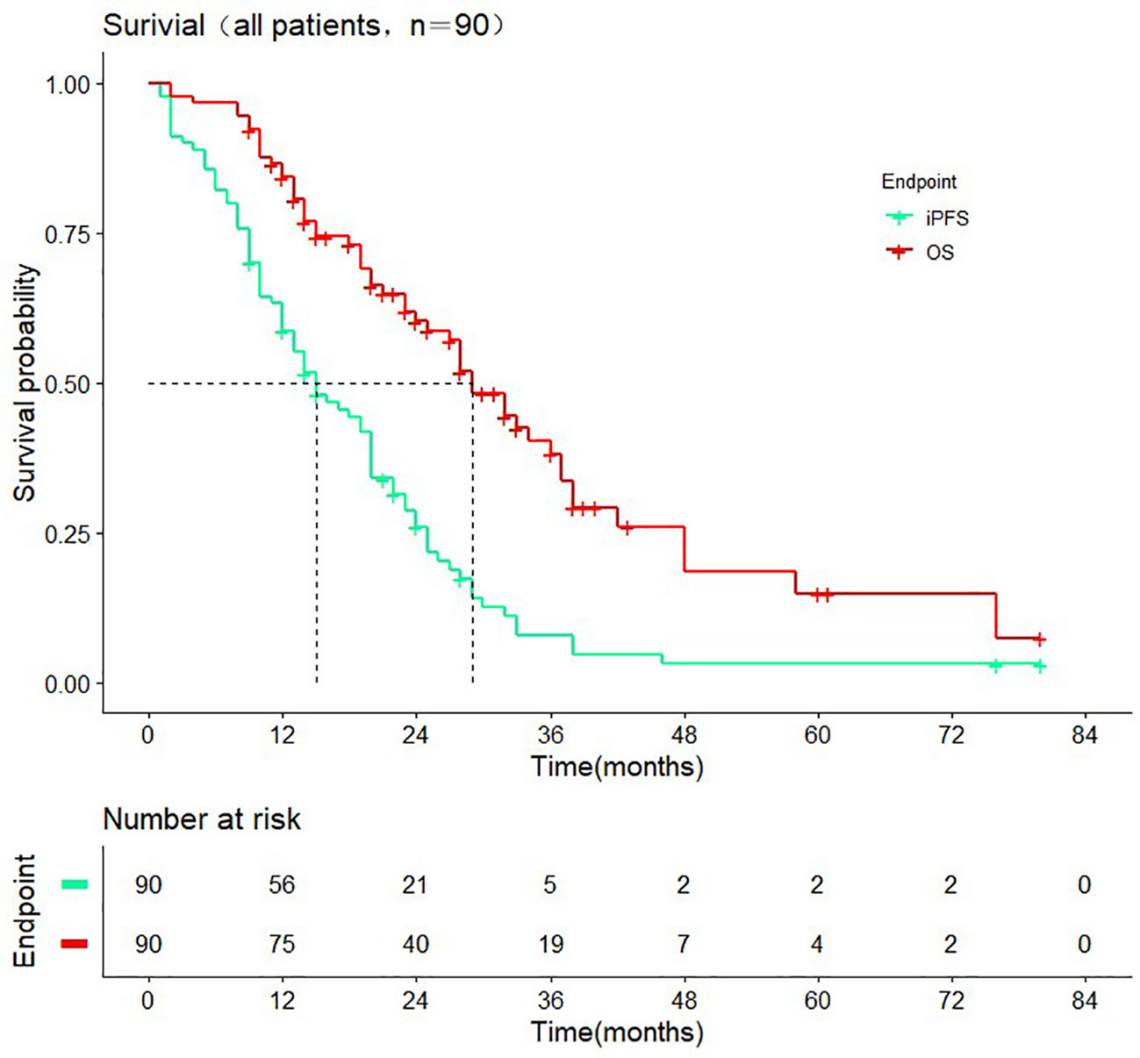
Intracranial progression-free-survival and overall survival in all patients.

The median follow-up time was 38.0 months (range 2.0-80.0 months). The median iPFS of enrolled patients was 15.0 months (95% CI: 10.2-19.7 months) median OS was 29.0 months (95% CI: 24.3-33.7 months) **(** **Figure 2**). As of the last follow-up, there were 31 and 45 cases of intracranial progression or death in the WBRT+SIB and SRS groups, respectively.

### 3.3 Subgroup analysis

**Figure 3:**
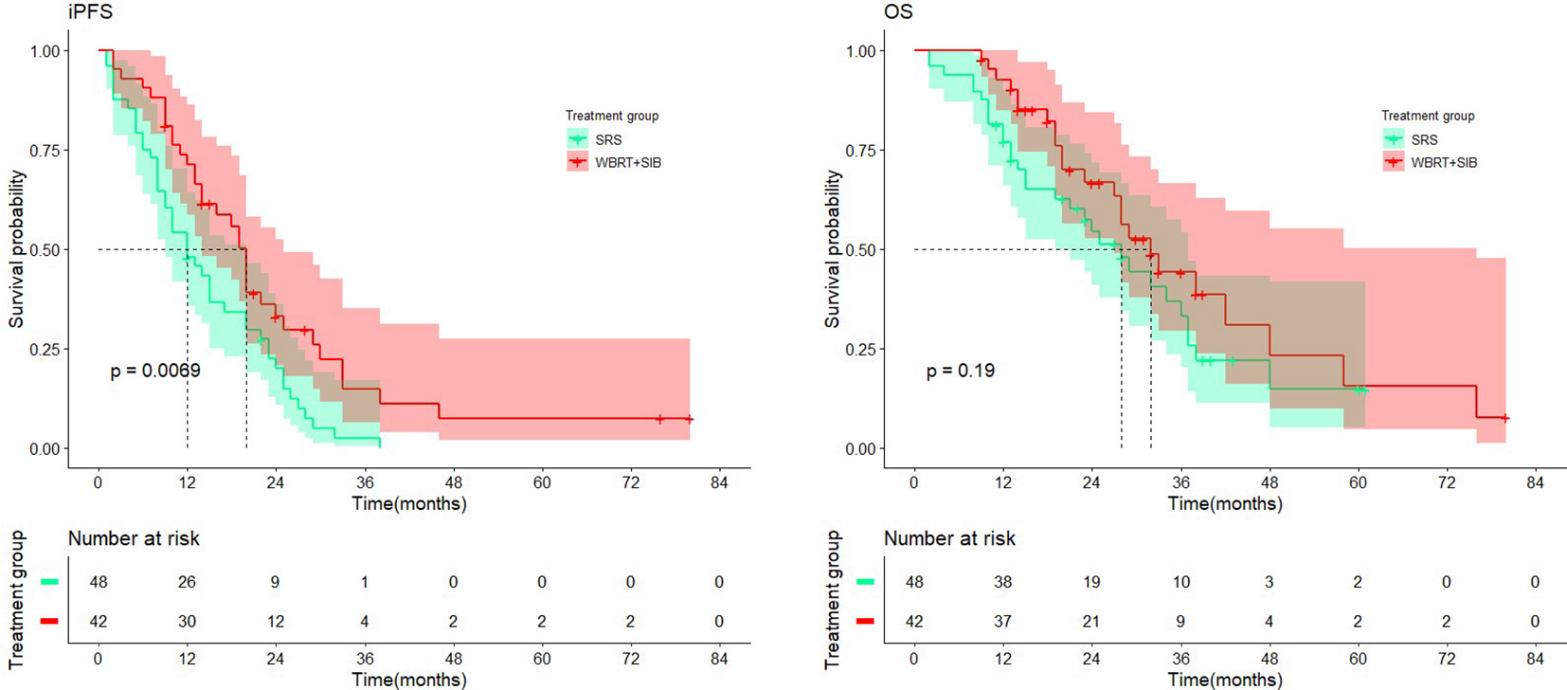
Comparison of intracranial progression-free-survival and overall survival in treatment cohort.

Except for the number of BMs (P < 0.001), there were no significant differences in other baseline characteristics between the two cohorts. Overall mortality was 54.7% in the WBRT+SIB cohort and 62.5% in the SRS cohort. Median iPFS was significantly longer in the WBRT+SIB cohort than in the SRS cohort (20.0 vs. 12.0 months,P= 0.0069, **Figure 3**), and median OS was also longer in the WBRT+SIB cohort (32.0 vs. 28.0 months P = 0.19, **Figure 3**), though, the difference in OS was not statistically significant.

### 3.4 Univariate/multivariate analysis

In univariate analysis, radiotherapy modality (P = 0.010) had the predictive value for iPFS. Histological status, Liver metastasis, Lymph node metastasis, BMI and Hypertension had the predictive value for OS. Factors which P ≤ 0.2 in univariate analysis were included in multifactorial analysis, Cox regression model analysis showed that the only independent prognostic factor for iPFS was Treatment group (**Table 2**). Sex, Liver metastasis and Lymph node metastasis are risk factors for NSCLC-BM (**Table 3**).

**Table 2:** Survival-related factors on iPFS in univariate/multivariate analysis.

◆Univariate analysis				○Multivariate analysis			
Pvalue	HR	Lower	Upper	Pvalue	HR	Lower	Upper
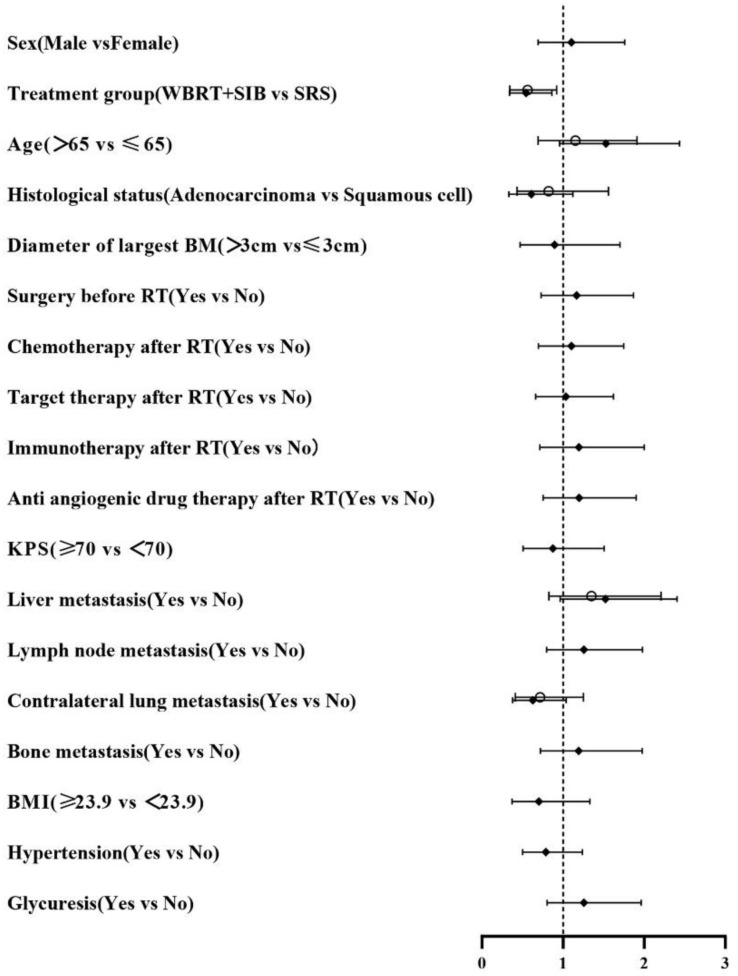	0.679 **0.010** **0.075** **0.112** 0.738 0.520 0.673 0.874 0.499 0.440 0.631 **0.070** 0.324 **0.070** 0.493 0.280 0.301 0.316	1.104 0.544 1.528 0.610 0.897 1.167 1.104 1.037 1.195 1.199 0.875 1.524 1.257 0.627 1.193 0.703 0.788 1.257	0.692 0.342 0.959 0.331 0.472 0.729 0.697 0.663 0.714 0.756 0.508 0.966 0.798 0.378 0.720 0.371 0.501 0.804	1.760 0.863 2.436 1.122 1.702 1.870 1.748 1.622 2.000 1.903 1.508 2.407 1.980 1.040 1.976 1.333 1.238 1.965	**0.023** 0.579 0.554 0.231 0.246	0.564 1.154 0.824 1.352 0.720	0.345 0.695 0.434 0.826 0.414	0.922 1.915 1.564 2.214 1.254

WBRT+SIB, Whole- Brain Radiation Therapy with Simultaneous Integrated Boost; SRS, Stereotactic Radiosurgery; BM, brain metastasis; RT, Radiotherapy; KPS, Karnofsky; BMI, Body mass index.Significance in bold was P < 0.2 in univariate analysis. In multivariate analysis, significance in bold is P< 0.05.

**Table 3:** Survival-related factors on OS in univariate/multivariate analysis.

	◆Univariate analysis				○Multivariate analysis			
Pvalue	HR	Lower	Upper	Pvalue	HR	Lower	Upper
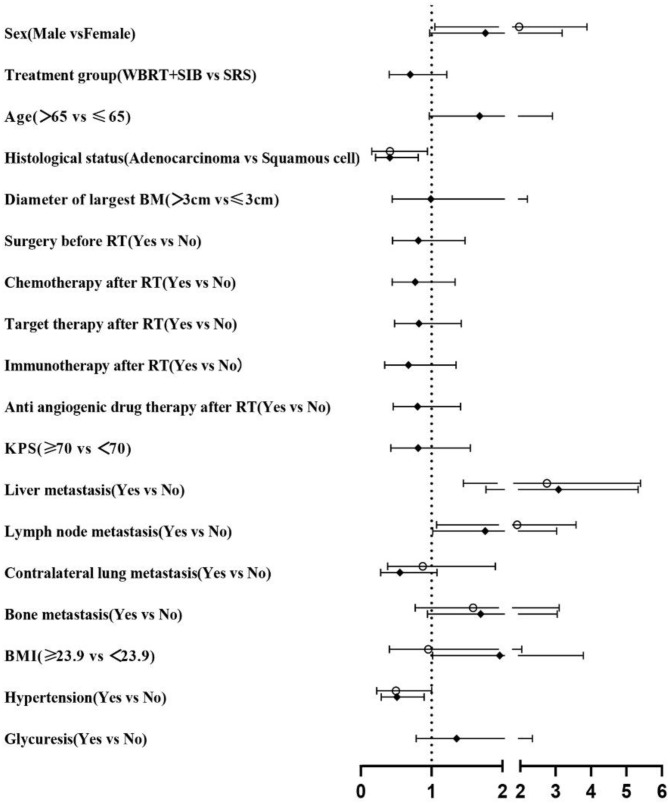	**0.062** 0.204 0.066 **0.010** 0.980 0.496 0.347 0.484 0.264 0.445 0.524 **<0.001** **0.043** **0.082** **0.078** **0.045** **0.019** 0.278	1.761 0.700 1.678 0.411 0.990 0.814 0.768 0.823 0.673 0.803 0.810 3.084 1.757 0.552 1.693 1.961 0.510 1.354	0.973 0.403 0.967 0.208 0.444 0.450 0.443 0.478 0.337 0.457 0.424 1.768 1.019 0.282 0.942 1.015 0.291 0.783	3.187 1.214 2.913 0.811 2.207 1.473 1.331 1.418 1.347 1.410 1.549 5.328 3.029 1.078 3.044 3.786 0.894 2.342	**0.020** 0.059 **0.001** **0.015** 0.912 0.134 0.921 0.088	2.138 0.496 2.924 2.080 0.960 1.670 1.039 0.580	1.129 0.240 1.535 1.156 0.464 0.853 0.488 0.310	4.052 1.026 5.568 3.745 1.985 3.267 2.212 1.085

WBRT+SIB, Whole- Brain Radiation Therapy with Simultaneous Integrated Boost; SRS, Stereotactic Radiosurgery; BM, brain metastasis; RT, Radiotherapy; KPS, Karnofsky; BMI, Body mass index. Significance in bold was P < 0.2 in univariate analysis. In multivariate analysis, significance in bold is P< 0.05.

### 3.5 Intracranial objective response rate

The WBRT+SIB and SRS groups had similar objective intracranial remission rates (objective response rate (ORR): 76.19% vs. 70.09%, P = 0.566) (disease control rate (DCR): 88.09% vs. 83.33%, P = 0.521) The brain CT/MRI at the third month after radiotherapy showed 2 cases of complete response (CR), 30 cases of partial response (PR), 5 cases of stable disease (SD), and 5 cases of progressive disease (PD) in the WBRT+SIB group and 1 case of CR, 33 cases of PR, 6 cases of SD, and 8 cases of PD in the SRS group (**Table 4**).

**Table 4:** Overall response.

	WBRT+SIB	SRS	Pvalue
ORR	32(76.19%)	34(70.09%)	0.566
DCR	37(88.09%)	40(83.33%)	0.521
CR	2 (4.76%)	1 (2.08%)	
PR	30(71.43%)	33(68.75%)	
SD	5 (11.90%)	6 (12.50%)	
PD	5 (11.90%)	8 (16.67%)	

WBRT+SIB, Whole- Brain Radiation Therapy with Simultaneous Integrated Boost; SRS, Stereotactic Radiosurgery; ORR, objective response rate; DCR, disease control rate; CR, complete response; PR, partial response; SD, stable disease; PD, progressive disease.

### 3.6 Toxicity

Post-radiotherapy-related toxicity according to the RTOG standard mainly included Nausea and vomiting, Leukopenia, Thrombocytopenia and CNS symptoms (including speech impairment, impaired consciousness, drowsiness, etc.) Acute radiation injury and late radiation injury as well as injury grading are shown in (**Table 5**).

**Table 5:** Comparison of adverse events in treatment cohort.

		Grade	WBRT+SIB(n=42)	SRS(n=48)
Acute radiation injury	Nausea/vomiting	0	19	27
		1	14	11
		2	7	9
		3	2	1
		4	0	0
	Leukopenia	0	27	32
		1	10	7
		2	5	8
		3	0	1
		4	0	0
	Thrombocytopenia	0	34	39
		1	7	7
		2	1	2
		3	0	0
		4	0	0
	CNS symptoms	0	33	37
		1	6	7
		2	2	4
		3	1	0
		4	0	0
Late radiation injury	CNS symptoms	0	22	35
		1	12	10
		2	5	2
		3	2	1
		4	1	0
		5	0	0

WBRT+SIB, Whole- Brain Radiation Therapy with Simultaneous Integrated Boost; SRS, Stereotactic Radiosurgery; CNS, central nervous system.

## 4 Discussion

This single-center retrospective study aimed to compare the effects of two radiotherapy modalities, WBRT+SIB and SRS, for the treatment of NSCLC-BM, with the primary study endpoints of iPFS and OS. The study results showed that compared to SRS, iPFS was significantly improved in the WBRT+SIB group (20.0 vs. 12.0 months, P = 0.0069). The difference in OS was not statistically significant (32.0 vs. 28.0 months, P = 0.19); patients with adenocarcinoma only tended to benefit in OS and iPFS compared to squamous cell carcinoma, but not statistically significant. The two groups had no significant difference in the current objective intracranial remission rate (76.19% vs. 70.09%, P = 0.566).

Current studies suggest that OS in patients with BMs under systemic therapy no longer appears to be limited by the control of intracranial lesions but instead had a more significant relationship with systemic disease progression (12). Our results also support the above view. In addition, we found relevant factors affecting OS of NSCLC-BM, including lymph node metastasis, bone metastasis, and hypertension.

The choice of radiotherapy modality when treating different kinds of BM patients is an issue that requires careful consideration. Yamamoto et al. (13) found that after SRS treatment, patients in the BM number 2-4 and 5-10 groups had the same OS. No difference in OS was found between BM number ≥10 and 2-9 groups (14). More and more evidence showed that SRS should no longer be limited to the number of BM. WBRT tends to withdraw from the mainstream of BM radiotherapy. What is more, WBRT is associated with poor cognitive function and decreased quality of survival. Theoretically, Radiation alters CNS barrier permeability in a time-dose-dependent manner (15–18). WBRT has a stronger ability to open up drug delivery barriers in the CNS than SRS, so it, combined with drugs, is more effective in treatment. Considering the long-term radiotherapy toxicity associated with WBRT, studies on Hippocampal Avoidance-Whole Brain Radiotherapy with simultaneous integrated boost(HA-WBRT+SIB)are increasing (19). Due to the highly economical and technical barriers of SRS/HA-WBRT+SIB, it i urgent to promote research on WBRT+SIB as a cost-effective approach.

The studies by Rodrigues et al. (20) and Du et al. (21) only included patients who received chemotherapy after radiotherapy and did not count patients treated with targeted drugs and ICIs. To be more clinically relevant, 84.44% of the included patients in our study were treated with scientific systemic drug therapy, including chemotherapy, targeted therapy, immunotherapy, anti-angiogenic drugs therapy, and supportive therapy after radiotherapy. Small cell lung cancer was also not included in this study. All these made the included patients have longer iPFS and OS (**Figure 2**). In general, the use of targeted drugs and ICIs is associated with a better prognosis. Although not indicated in the figure, we found that whether the patients received ICIs or targeted therapy iPFS (ICIs compared cohort: p = 0.49 Targeted therapy compared cohort: p = 0.84) and OS (ICIs compared cohort: p = 0.25 Targeted therapy cohort: p = 0.48) were not statistically different (**Supplementary Figures 1**–**4**). 78% of patients who did not receive targeted therapy received other drugs. In ICIs compared cohort, this rate was 94%. Other drugs may have obscured the survival benefit of single targeted drugs or ICIs therapy. We may conclude that WBRT+SIB is preferable to SRS under the standard treatment mode of radiotherapy plus drugs for NSCLC-BM patients. Regarding radiotherapy-related toxicity, a prospective study by Zhong et al. (22) demonstrated the safety and efficacy of WBRT+SIB, which was similar to the results of the radiotherapy-related toxicity assessment in this study, i.e., no significant difference was seen between WBRT+SIB and SRS in recent radiotherapy-related toxicity.

A study from the SEER database of lung cancer by Hao (23) et al. showed that distant liver/bone/lymph node metastases, higher T and N stages were risk factors for NSCLC-BM. Our study only found that Sex, Liver metastasis and Lymph node metastasis were independent prognostic factors for NSCLC-BM. Considering that the participants in our study all received brain radiotherapy, this may account for the difference between our conclusion and Hao et al. Further studies are needed to confirm our conclusions.

This study has several limitations; First, as a retrospective study, we were biased and enrolled a small number of patients. Then, our study lacked drug side effects (e.g.,immune-related adverse events, Bleeding risk, skin reactions, etc.) and did not document the long-term cognitive function and quality of life changes in patients after treatment. Moreover, most patients received multiple drug combinations after radiotherapy, which may have masked the effect of single drug classes on survival.

In conclusion, the results of this study suggest that Radiotherapy modality is a crucial and independent prognostic factor in patients with NSCLC-BM, and WBRT+SIB seems to be associated with a more favorable prognosis compared to SRS. Presently, the treatment of drugs such as mannitol and hormone to reduce the toxicity of radiotherapy is more and more standardized, and the probability of short-term toxicity is less and less. Although not described in detail in this study, we must consider the question of long-term cognitive function and quality of life decline associated with WBRT. Although there is no difference in OS between WBRT+SIB and SRS, as we all know. Compare to WBRT+SIB,Whether the advantages of repeatability and security of SRS can offset the disadvantages of economy and technology still, need to be considered comprehensively. In the future, we should focus on finding a balance in treating BM by making trade-offs between intracranial control, management of systemic progression, and neurocognitive decline in patients. However, prospective, large-sample randomized controlled trials are needed to validate our results.

## Data availability statement

The original contributions presented in the study are included in the article/**Supplementary Material**. Further inquiries can be directed to the corresponding author.

## Ethics statement

The studies involving humans were approved by The Affiliated Cancer Hospital of Zhengzhou University. The studies were conducted in accordance with the local legislation and institutional requirements. The ethics committee/institutional review board waived the requirement of written informed consent for participation from the participants or the participants’ legal guardians/next of kin because This study was approved by the ethics committees of the Affiliated Cancer Hospital of Zhengzhou University & Henan Cancer Hospital, Zhengzhou, China. Due to the retrospective nature of the study and because no patient specimens were used, the requirement for informed consent was waived by the ethics committees.

## Author contributions

XD and LY designed this study and analyzed the data. XD collected the data and wrote the manuscript. HY, YH, JC and YL assisted in collecting data and correcting the manuscript. All authors contributed to the article and approved the submitted version.

## Supplementary material

Supplementary Figure 1 Comparison of intracranial progression-free-survival in ICIs compared cohort. ICIs, immune checkpoint inhibitors.

Supplementary Figure 2 Comparison of intracranial progression-free-survival in targeted therapy compared cohort.

Supplementary Figure 3 Comparison of overall survival in ICIs compared cohort. ICIs, immune checkpoint inhibitors.

Supplementary Figure 4 Comparison of overall survival in targeted therapy compared cohort.
