# Supplementary figures and images for: Choice of radiotherapy modality for the combined treatment of non-small cell lung cancer with brain metastases: whole-brain radiation therapy with simultaneous integrated boost or stereotactic radiosurgery
Source: Front Oncol. 2023 Sep 22;13:1220047. doi: 10.3389/fonc.2023.1220047 (PMC10556697; doi:10.3389/fonc.2023.1220047)

Strata No Yes

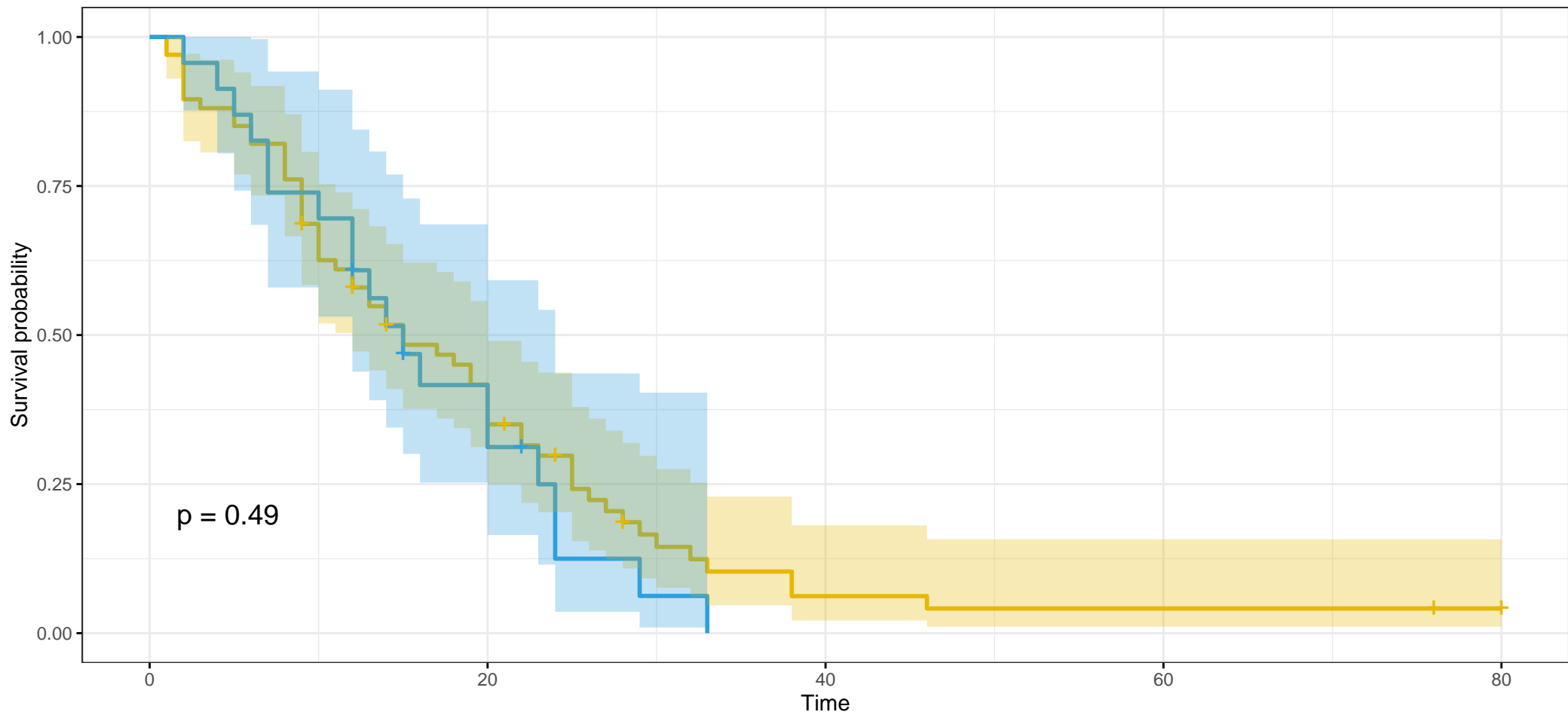

Number at risk

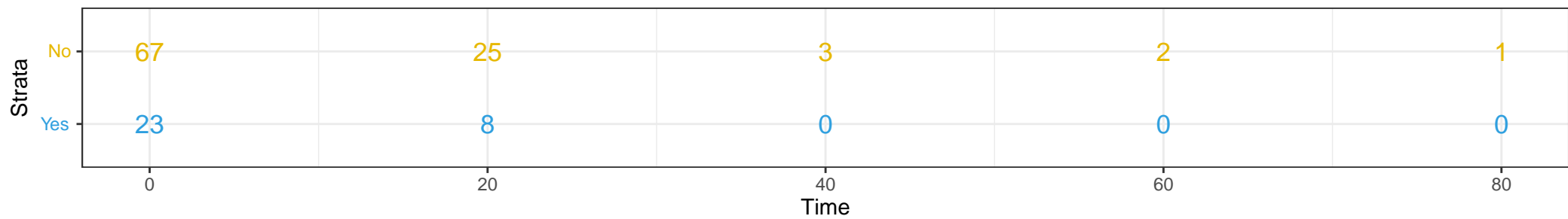

Supplement: Supplementary Figure 1 — Comparison of intracranial progression-free-survival in ICIs compared cohort. ICIs, immune checkpoint inhibitors. [file DataSheet_1.pdf]

Strata    No    Yes

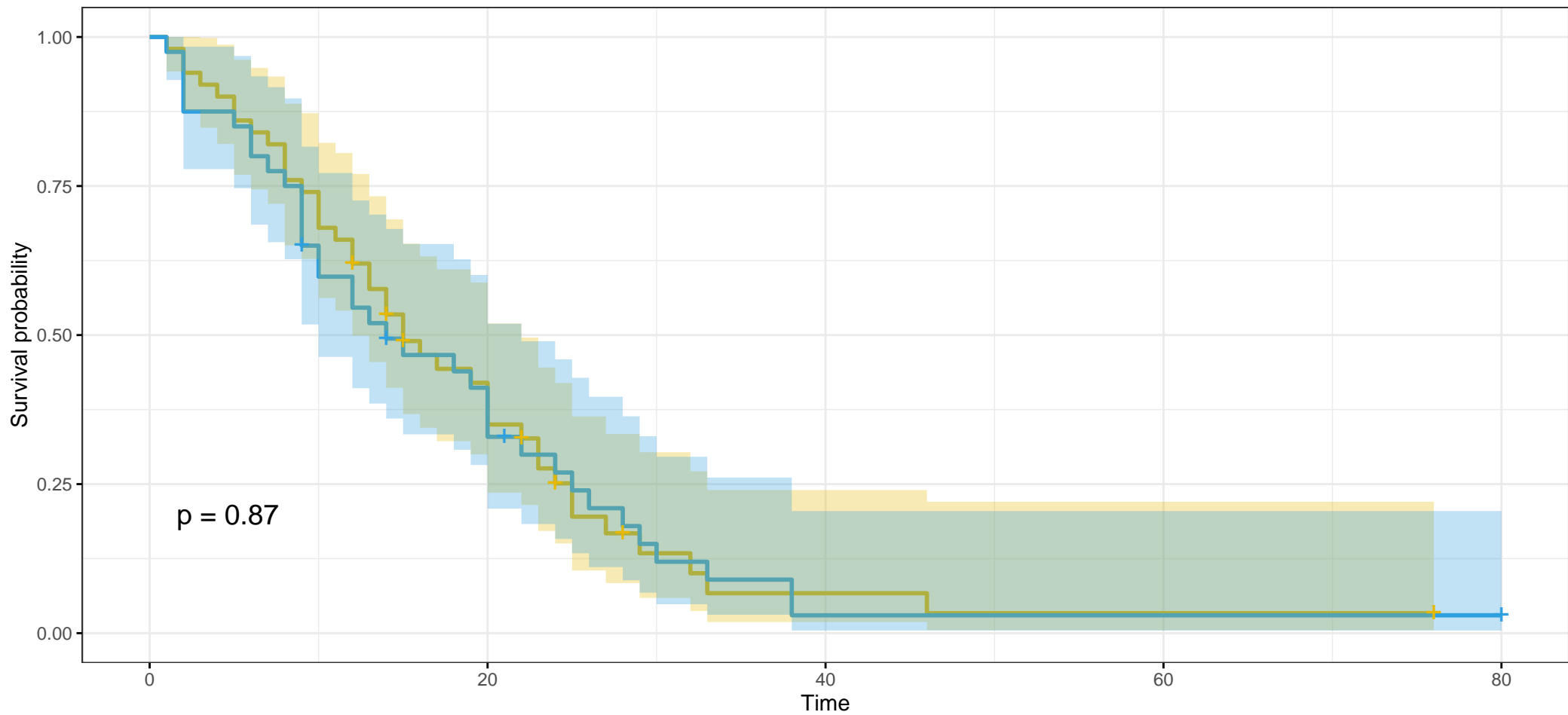

Number at risk

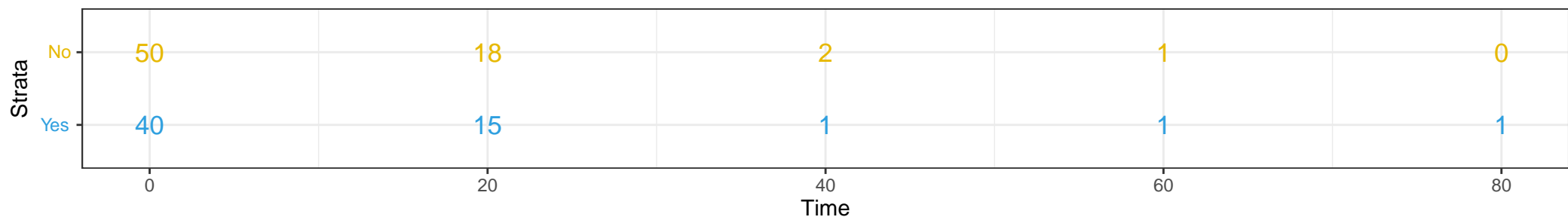

Supplement: Supplementary Figure 2 — Comparison of intracranial progression-free-survival in targeted therapy compared cohort. [file DataSheet_2.pdf]

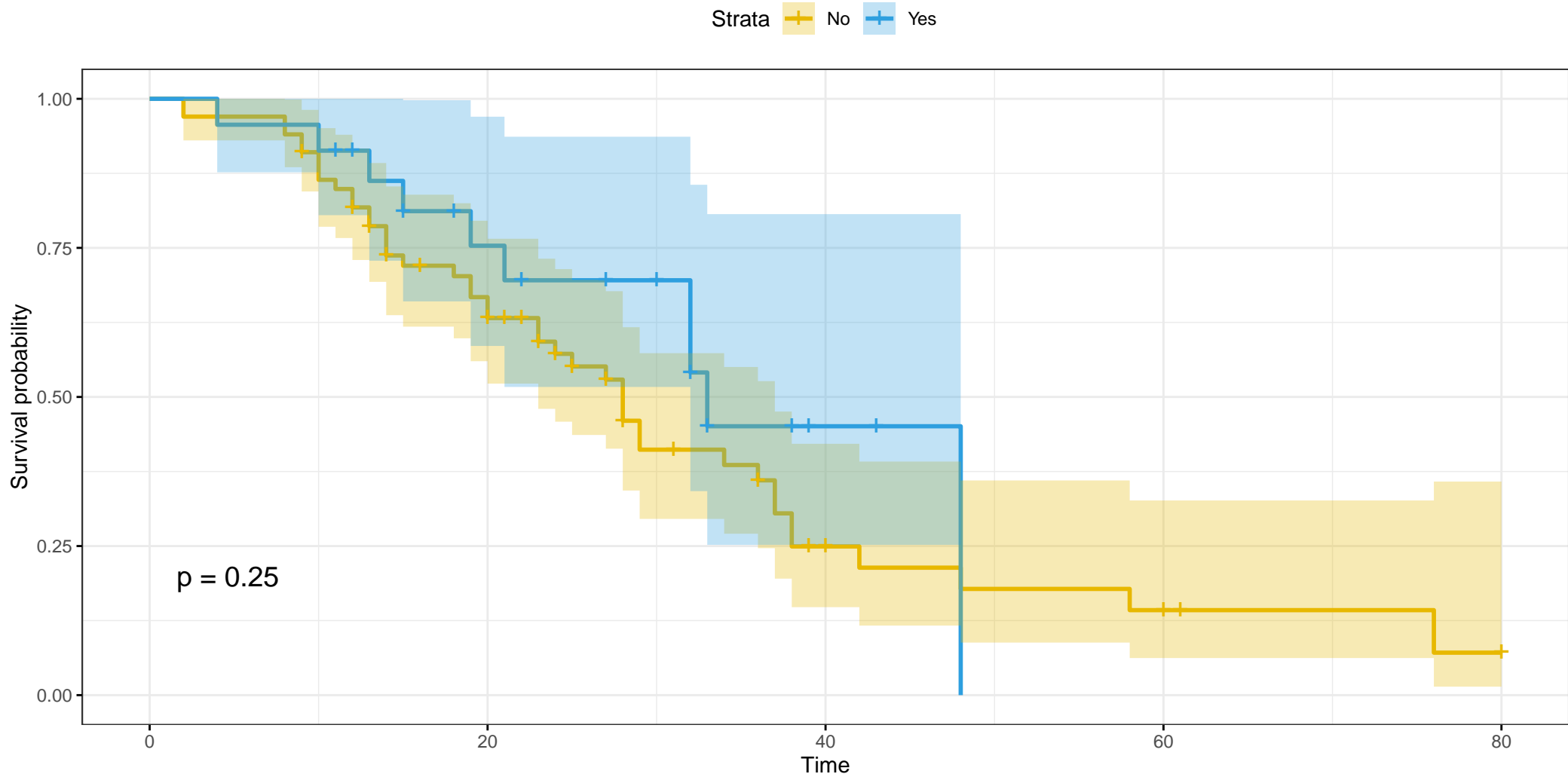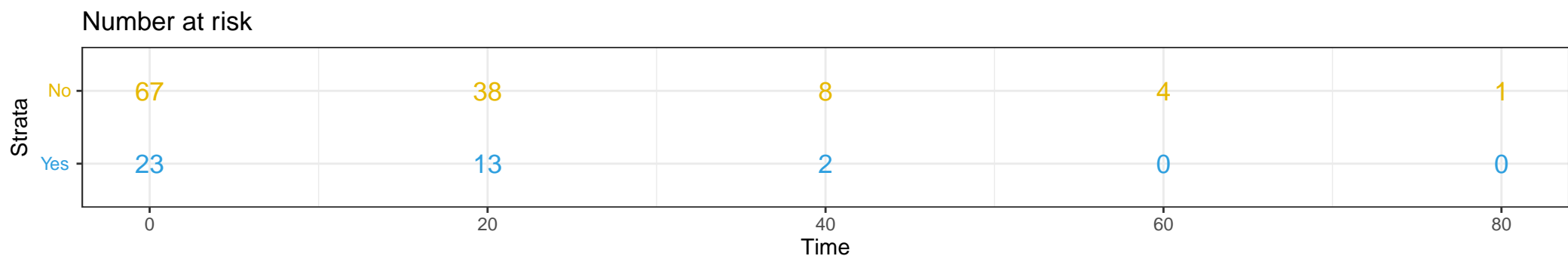

Supplement: Supplementary Figure 3 — Comparison of overall survival in ICIs compared cohort. ICIs, immune checkpoint inhibitors. [file DataSheet_3.pdf]

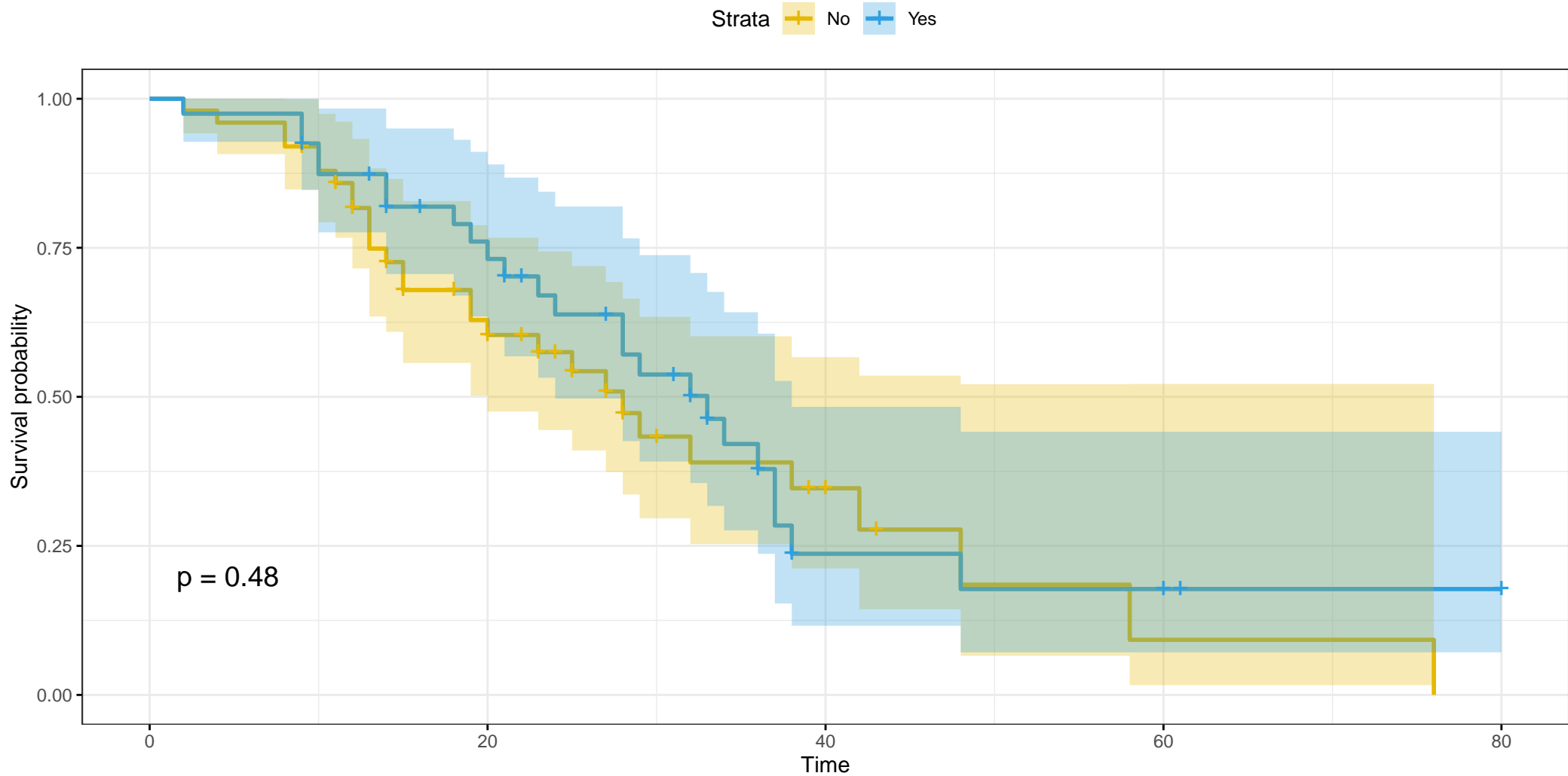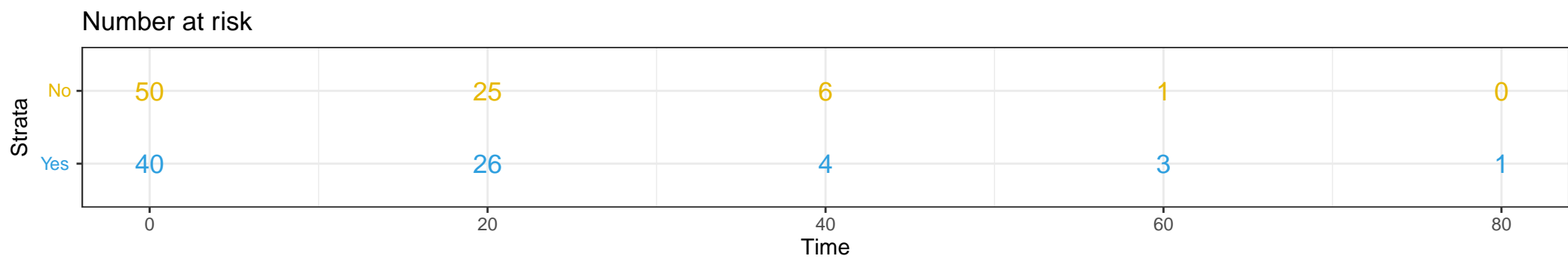

Supplement: Supplementary Figure 4 — Comparison of overall survival in targeted therapy compared cohort. [file DataSheet_4.pdf]
